# Supplementary material for: Sex differences in bile acid homeostasis and excretion underlie the disparity in liver cancer incidence between males and females
Source: eLife. 2025 Dec 29;13:RP96783. doi: 10.7554/eLife.96783 (PMC12747522; doi:10.7554/eLife.96783)
Supplement: Figure 2—source data 2. [file elife-96783-fig2-data2.docx]

| TF Name | Density  ratio | P-Value |
| --- | --- | --- |
| PPARγ | 1.30126 | 6.3527E-11 |
| TCF3 | 1.33458 | 7.2023E-11 |
| TCF4 | 1.27146 | 5.5608E-9 |
| p73 | 1.30219 | 1.1841E-5 |
| LRH1 | 1.1917 | 2.3061E-5 |
| AR | 1.3065 | 2.7733E-5 |
| FOXA2 | 1.21861 | 6.526E-4 |
| MEF2A | 1.35309 | 0.00153 |
| MEF2C | 1.34771 | 0.0016 |
| TBR2 (EOMES) | 1.15298 | 0.00168 |
| TR4 | 1.45287 | 0.00195 |
| GATA3 | 1.95407 | 0.00351 |
| c-JUN | 1.20405 | 0.00491 |
| STAT1 | 1.17336 | 0.00505 |
| RXRa | 1.42981 | 0.00512 |
| STAT5a | 1.19101 | 0.00557 |
| FOXA1 | 1.53342 | 0.00674 |
| STAT4 | 1.42981 | 0.00707 |
| NRF2 | 3.06387 | 0.00959 |
| KLF4 | 1.08893 | 0.01000 |
