# Supplementary material for: Sex differences in bile acid homeostasis and excretion underlie the disparity in liver cancer incidence between males and females
Source: eLife. 2025 Dec 29;13:RP96783. doi: 10.7554/eLife.96783 (PMC12747522; doi:10.7554/eLife.96783)
Supplement: Figure 2—source data 3. [file elife-96783-fig2-data3.docx]

| TF Name | Density ratio | P-Value |
| --- | --- | --- |
| USF1 | 3.10842 | 6.0846E-4 |
| GATA1 | 1.26352 | 0.00179 |
| THAP1 | 3.38041 | 0.00226 |
| FOXO1 | 2.6577 | 0.00247 |
| E2F4 | 1.53201 | 0.00262 |
| SP1 | 6.29455 | 0.00267 |
| E2F6 | 1.79844 | 0.00439 |
| GR | 1.31386 | 0.00484 |
| ZFX | 1.2357 | 0.00614 |
| NKX2-5 | 1.72517 | 0.00633 |
| USF2 | 2.09818 | 0.00675 |
| ZBTB33 | 1.76247 | 0.0091 |
| YY1 | 1.29119 | 0.01000 |
| ESR1 | 1.72159 | 0.01000 |
