# Supplementary material for: Sex differences in bile acid homeostasis and excretion underlie the disparity in liver cancer incidence between males and females
Source: eLife. 2025 Dec 29;13:RP96783. doi: 10.7554/eLife.96783 (PMC12747522; doi:10.7554/eLife.96783)
Supplement: Figure 3—source data 1. [file elife-96783-fig3-data1.docx]

| Cohorts | Platform | Number of  patients | Source |
| --- | --- | --- | --- |
| Fudan | Affymetrix U133A2 | 247 | GEO, GSE14520 |
| Samsung | RNA-seq  Illumina HumanHT-12 V4.0 | 240 | GEO, GSE36376 |
| TCGA -LIHC | RNA seq | 373 | https://xenabrowser.net/ |
| Korea | Illumina V2, V4 | 188 | GEO, GSE16757, GSE43619 |
| Modena | Agilent-014850 | 78 | GEO, GSE54236 |
