# Supplementary material for: Sex differences in bile acid homeostasis and excretion underlie the disparity in liver cancer incidence between males and females
Source: eLife. 2025 Dec 29;13:RP96783. doi: 10.7554/eLife.96783 (PMC12747522; doi:10.7554/eLife.96783)
Supplement: Figure 5—source data 1. [file elife-96783-fig5-data1.docx]

|  | DKO Male | DKO Female |
| --- | --- | --- |
| Serum | n=4 | n=3 |
| Amidation |  |  |
| % Amidation | 73.67% | 77.66% |
| % G-Amidation | 0.14% | 0.11% |
| % T-Amidation | 73.53% | 77.55% |
| BA Hydroxylation |  |  |
| % Mono-OH | 0.15% | 0.27% |
| % Di-OH | 13.53% | 12.19% |
| % Tri-OH | 81.78% | 82.51% |
| BA Composition |  |  |
| % LCA | 0.15% | 0.27% |
| % UDCA | 0.39% | 0.47% |
| % CDCA | 0.79% | 1.01% |
| % DCA | 12.09% | 10.41% |
| % HDCA | 0.19% | 0.23% |
| % MDCA | 0.07% | 0.06% |
| % CA | 69.56% | 65.50% |
| % MCA | 12.21% | 17.00% |
| % HCA | 0.01% | 0.00% |
|  |  |  |
| Primary BA (μM) | 60.10 | 36.27 |
| Secondary BA (μM) | 12.68 | 7.39 |
| Primary/Secondary | 5.62 | 5.36 |
| 12α/non12α | 5.22 | 4.03 |
| % Sulfation | 1.72% | 2.66% |
| Hydrophobicity Index | 0.029 | -0.029 |
