# Supplementary material for: Sex differences in bile acid homeostasis and excretion underlie the disparity in liver cancer incidence between males and females
Source: eLife. 2025 Dec 29;13:RP96783. doi: 10.7554/eLife.96783 (PMC12747522; doi:10.7554/eLife.96783)
Supplement: Figure 5—source data 2. [file elife-96783-fig5-data2.docx]

|  | DKO Male | DKO Female |
| --- | --- | --- |
| Urine | n=4 | n=3 |
| Amidation |  |  |
| % Amidation | 79.62% | 20.52% |
| % G-amidation | 0.20% | 0.52% |
| % T-amidation | 93.32% | 37.81% |
| BA Hydroxylation |  |  |
| % Mono-OH | 0.01% | 0.78% |
| % Di-OH | 3.51% | 15.48% |
| % Tri-OH | 96.17% | 76.71% |
| BA Composition |  |  |
| % LCA | 0.01% | 0.78% |
| % UDCA | 1.40% | 0.00% |
| % CDCA | 0.21% | 1.20% |
| % DCA | 1.67% | 0.64% |
| % HDCA | 0.00% | 0.00% |
| % MDCA | 0.24% | 13.64% |
| % CA | 91.17% | 37.82% |
| % MCA | 4.86% | 34.90% |
| % HCA | 0.14% | 4.00% |
|  |  |  |
| % Primary BA | 96.36% | 80.00% |
| % Secondary BA | 3.64% | 20.00% |
| Primary/Secondary | 33.52 | 4.13 |
| 12a/non12a | 2.21 | 0.63 |
| % Sulfation | 79.62% | 20.52% |
| Hydrophobicity Index | -0.09 | -0.13 |
|  |  |  |
