# Supplementary material for: Sex differences in bile acid homeostasis and excretion underlie the disparity in liver cancer incidence between males and females
Source: eLife. 2025 Dec 29;13:RP96783. doi: 10.7554/eLife.96783 (PMC12747522; doi:10.7554/eLife.96783)
Supplement: Supplementary file 1. [file elife-96783-supp1.docx]

**Supplemental File 1. Primer sequences used**

| Gene  Name | Forward Primer  5’ to 3’ | Reverse Primer  5’ to 3’ |
| --- | --- | --- |
| *36B4* | AGATGCAGCAGATCCGCAT | GTTCTTGCCCATCAGCACC |
| *Ccnd1* | CCTCCTTCTGCACACATTTGA | GCTGCGAAGTGGAAACCATC |
| *Cyp7a1* | CAGGGAGATGCTCTGTGTTCA | AGGCATACATCCCTTCCGTGA |
| *Cyp8b1* | AAGGCTGGCTTCCTGAGCTT | AACAGCTCATCGGCCTCATC |
| *Cyp27a1* | CCTACATCCATTCGGCTCT | CCAGGGCAATCTCATACTTC |
| *Cyp7b1* | GACGATCCTGAAATAGGAGCACA | AATGGTGTTTGCTAGAGAGGCC |
| *Cyp2c70* | TGGCTTTCTCAGCAGGAAGAA | AACTGGCTTGGTGTCGATGT |
| *Abcb11* | CACACAAAGCCCCTACCAGT | CCAAGAGGCAGCTATCAGGC |
| *Slc10a1* | GGTGCCCTACAAAGGCATTA | GTTGCCCACATTGATGACAG |
| *Abcb1* | ACTCGGGCGCAGAACTTTGA | GCACCAAAGACAACAGCAGA |
| *Abcc2* | GCACTGTAGGCTCTGGGAAG | CATTTCCAAGTCTGGGAGGA |
| *Abcc3* | GCAGAGACAGGCAATGTGAA | GAAAGCTGACAGCATGACCA |
| *Sult2a1* | CCTCAAAAGAAATGTTCTAATCGGA | CTTTCATGGCTTGGAAAGAGCTGTA |
| *Esr1* | GACAAGCGGCGTAAAAG | CATTATGGGGTCTGGTCCTG |
| *Cps1* | AGGCACCACCATTACCTCAG | CGGGTTCATCAGGACTGTTT |
| *Ass1* | ACACCTCCTGCATCCTCGT | GCTCACATCCTCAATGAACACCT |
| *Asl* | CTAGAGGTACAGAAGCGG | TGCTGTTGAGAGTGATGG |
| *Arg1* | CTCCAAGCCAAAGTCCTTAGAG | AGGAGCTGTCATTAGGGACATC |
| *Otc* | TTTGGGTGTGAATGAAAGTCTC | TGATGATTGGGATGGATGCT |
| *Gls2* | CCGTGGTGAACCTGCTATTT | TGCGGGAATCATAGTCCTTC |
| *Gdh* | GGCCGATTGACCTTCAAATA | TCCTGTCCTGGAACTCTGCT   \| *Cyp7b1* \| Mouse \| GACGATCCTGAAATAGGAGCACA \| AATGGTGTTTGCTAGAGAGGCC \| \| --- \| --- \| --- \| --- \| \| *Cyp2c70* \| Mouse \| TGGCTTTCTCAGCAGGAAGAA \| AACTGGCTTGGTGTCGATGT \| |
| *Glul* | CAGGCTGCCATACCAACTTCA | TCCTCAATGCACTTCAGACCAT |
|  |  |  |
| ChIP-PCR |  |  |
| *Cyp7a1* | TTCCCCATCAGTTTGCAGGT | ACCTCCCAAATCTGCGTCTT |
| *Cyp8b1* | GAGGACCCAGAAACAGGTGG | TGTCTACTCCCTACTGGGGC |
| *Ldlr* | CAGAGCCATCGTAGTGGACC | TTGGTCTATCACCGACACCC |
| *Pgr* | TGATGTGGTCTATGCAGGGC | TGGTGCTCTTTACATGGTAGTTG |
